# Coordinated vigilance provides evidence for direct reciprocity in coral reef fishes

**DOI:** 10.1038/srep14556

**Published:** 2015-09-25

**Authors:** Simon J. Brandl, David R. Bellwood

**Affiliations:** 1 ARC Centre of Excellence for Coral Reef Studies, James Cook University, Townsville, Queensland 4811, Australia; 2 College of Marine and Environmental Sciences, James Cook University, Townsville, Queensland 4811, Australia

## Abstract

Reciprocity is frequently assumed to require complex cognitive abilities. Therefore, it has been argued that reciprocity may be restricted to animals that can meet these demands. Here, we provide evidence for the potential presence of direct reciprocity in teleost fishes. We demonstrate that in pairs of coral reef rabbitfishes (f. Siganidae), one fish frequently assumes an upright vigilance position in the water column, while the partner forages in small crevices in the reef substratum. Both behaviours are strongly coordinated and partners regularly alternate their positions, resulting in a balanced distribution of foraging activity. Compared to solitary individuals, fishes in pairs exhibit longer vigilance bouts, suggesting that the help provided to the partner is costly. In turn, fishes in pairs take more consecutive bites and penetrate deeper into crevices than solitary individuals, suggesting that the safety provided by a vigilant partner may outweigh initial costs by increasing foraging efficiency. Thus, the described system appears to meet all of the requirements for direct reciprocity. We argue that the nature of rabbitfish pairs provides favourable conditions for the establishment of direct reciprocity, as continuous interaction with the same partner, simultaneous needs, interdependence, and communication relax the cognitive demands of reciprocal cooperation.

Cooperation is widespread among animals^1^, and it is now widely accepted that cooperation is also present among unrelated individuals^2^,^3^,^4^,^5^. In this context, reciprocity or ‘reciprocal altruism’, which involves a costly action beneficial for another individual, based on an expected future payoff through reciprocation, has garnered particular interest^2^,^6^,^7^. This interest has arisen from the notion that cooperative individuals should be prone to exploitation by their respective partners if the latter defects after having received help^8^, therefore leading to fitness declines in cooperating individuals. However, it has been suggested that reciprocity can be evolutionarily stable, even if modelled under an iterated prisoner’s dilemma (IPD), in which both players employ a strategy called ‘tit-for-tat’^6^,^9^. The IPD and several other game-theoretical models have subsequently provided frameworks for the evaluation of reciprocity in animals and throughout the last few decades, the presence of reciprocity has been suggested in fishes^10^,^11^,^12^,^13^,^14^, birds^15^,^16^,^17^, and mammals^18^,^19^,^20^,^21^,^22^.

However, almost all reported occurrences of direct reciprocity in animals have now been challenged^23^, as many aspects of reciprocity are thought to require a suite of complex cognitive abilities. This includes the recognition of individual partners, the capacity to recall their previous actions, or the ability to make intentional investments under the expectation that it will entail a future reward^24^,^25^. For this reason, it has been questioned whether direct reciprocity exists in animals which are assumed to lack complex social and cognitive skills^23^,^26^,^27^,^28^,^29^,^30^, and most evidence of direct reciprocity to date is confined to a few cases in birds and mammals^18^,^20^,^22^,^31^. Yet, in response to these criticisms, it has also been posited that most of the cognitively demanding actions of reciprocity stem predominantly from the theoretical framework in which reciprocity is investigated^7^,^31^,^32^,^33^. Specifically, many aspects of game-theoretical models such as tit-for-tat in the IPD have been questioned over the last two decades^34^,^35^. Most recently, the discrete time structure of the IPD and other models, as well as their incapacity to allow for the exchange of information among partners during cooperative interactions, have been identified as a major weakness of these models^36^,^37^. These weaknesses undermine our capacity to adequately judge whether or not reciprocity is present^38^, particularly given the often emphasized need for continuous information exchange among cooperating partners in a natural setting^13^,^17^,^39^.

Teleost fishes have contributed substantially to the debate about cooperation and reciprocity in animals, and a variety of systems have been discussed in the context of the reciprocal exchange of commodities. This includes 1) egg-trading in hermaphroditic hamlets (f. Serranidae), which describes the exchange of costly eggs for fertilization by the partner^12^,^40^, 2) helping behaviour in cooperatively breeding cichlids (f. Cichlidae), which involves the trading of resource access against the provision of brood care and territorial defence (helpers ‘pay-to-stay’^41^,^42^), 3) interspecific cleaning behaviour by pairs of cleaner wrasses (f. Labridae), which involves the removal of parasites from client fishes^2^,^43^,^44^, and 4) predator inspection in sticklebacks (f. Gasterosteidae) and other small fishes^1^,^45^, which involves a pair of fishes approaching a predator in order to assess the threat arising from its presence, for which the presence of a partner provides a safer situation than if the fish were to approach the predator alone^13^,^45^. While most of these systems were initially identified as cases of reciprocity, subsequent assessments and syntheses have argued that they are based on pseudo-reciprocity and by-product mutualism rather than direct reciprocity^23^,^29^,^46^,^47^,^48^, invoking the limited potential of teleost fishes to cope with the cognitive and social demands of reciprocity. However, there is now substantial evidence for many complex social processes in fishes^49^,^50^,^51^,^52^,^53^,^54^, including image scoring^43^, punishment^55^, pre-emptive appeasement^41^,^42^, or partner preference^56^. Thus, teleost fishes appear to provide a promising group for the investigation of reciprocity^31^,^50^.

Rabbitfishes (f. Siganidae) are an Indo-Pacific family of teleost fishes, which includes 28 species and is separated in two major groups^57^. The first group consists of predominantly schooling species, which are drab-coloured and commonly associated with mangrove and estuarine habitats, while the second group consists of species which occur mostly in stable pairs, are brightly coloured, and are commonly associated with coral reefs^58^,^59^. In the latter group, the presence of same-sex pairs has spurred research exploring the potential ecological role of pairing behaviour in this family, and it has been proposed that their foraging habits, which entail the penetration of cracks and crevices in the reef matrix, may necessitate the presence of a vigilant partner^60^,^61^. Consequently, it has recently been suggested (based on photographic evidence) that pair members may cooperate in order to achieve high levels of vigilance during foraging^62^. However, the potential presence of cooperation among pair members in rabbitfishes has not been evaluated quantitatively and individual costs and benefits are unknown, undermining our ability to judge whether vigilance behaviour in pairing rabbitfishes may be based on reciprocity.

The goal of the present study, therefore, was to quantify the major aspects of pairing behaviour in rabbitfishes, and to assess whether the behaviour exhibited by rabbitfish pairs may satisfy the basic requirements of reciprocal cooperation, such as reciprocal alternation between feeding bouts and a costly investment by one individual that directly benefits the partner.

## Materials & Methods

### Data collection

All fieldwork was conducted on coral reefs around Lizard Island, a granitic mid-shelf island in the northern Great Barrier Reef, Australia (14°40′08″S 145°27′34″E). Four different species of pairing rabbitfishes were considered (*Siganus corallinus, S. doliatus, S. puellus, and S. vulpinus*), as they represented the most abundant pairing species in the study area (Big Vicki’s Reef). The four examined species differ in their dietary preferences, with *S. corallinus* and *S. doliatus* feeding predominantly on red corticated and red filamentous algae, while *S. puellus* feeds mainly on sponges, and *S. vulpinus* predominantly on cyanobacteria^63^. However, all species are similar in their foraging behaviours, as all four species have been reported to exploit cryptic reef micro-habitats such as cracks and crevices in the substratum^61^. In addition, all four species are strongly pair-forming, with approximately 80% of all adult individuals occurring in pairs in *S. corallinus, S. doliatus*, and *S. vulpinus*, and approximately 70% in *S. puellus*^59^.

**Figure 1:**
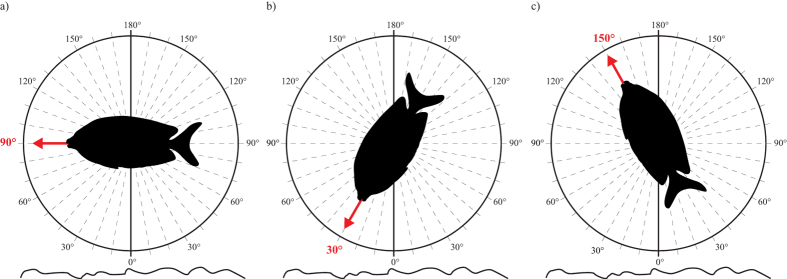
Schematic description of the assessment of angles exhibited by rabbitfishes. Angles were assessed based on a vertical line perpendicular to the substratum using a protractor superimposed on the screen. 90° denotes a horizontal position parallel to the substratum (**a**), while 30° mark a head-down position (**b**), and 150° mark a head-up position (**c**). The head-up position (ranging from ~90° to 180°) was identified as a vigilance position. Illustrations are original drawings by Simon J. Brandl.

Haphazardly encountered pairs of the four species were followed while SCUBA diving and video footage of their behaviour was collected for a period of 12 minutes for each pair (using Sony DCR-SR300E camcorders). Care was taken to sample different sites on the reef in order to avoid re-sampling of the same pairs. For each pair, the size was estimated, and video recording commenced one minute after the fish were observed to feed, in order to prevent behavioural biases due to the presence of the observer (foraging was interpreted as a sign that fish had acclimatized to the observer). Observers aimed to keep a constant distance from the recorded fish; video sequences in which the distance to the fish resulted in unreliable examination of fish behaviour or obvious behavioural responses to the observer were discarded from the analyses. Videos were collected during three different times of day (0600 to 1000 h; 1000 to 1400 h; 1400 to 1800 h) and subsequently analysed in 5-second intercepts. Specifically, videos were paused every five seconds to determine the current behaviour of the partners as a point measure. For every 5-second point intercept at which both members of the pair were visible in the video, the angles of both individuals (*θA* and *θB*) relative to a vertical line perpendicular to the substratum were measured using a protractor that was superimposed on the computer screen (Fig. 1). Angles were assessed quantitatively to describe a conspicuous behaviour, henceforth termed ‘vigilance position’, in which one individual was found hovering high above the substratum with its head oriented upwards (Fig. 1c). In addition, the fish’s height above the substratum (cm), the distance between pair members (cm), the complexity of the surrounding microhabitat (1 = lowest complexity to 5 = highest complexity), and the behaviour of individuals (swimming, foraging, hovering, or displaying) were recorded. These parameters were recorded because of their potential influence on the behaviour exhibited by the partners (for instance, higher complexity of the surrounding environment may reduce the threat arising from predation through the provision of shelter^64^). Swimming was defined as active, directional movements, while foraging was defined as at least one of the two individuals engaged in active feeding (i.e. biting from the substratum). Hovering entailed both individuals being stationary and motionless (similar to the vigilance position), and displaying was noted when individuals engaged in displays towards other pairs of the same species. The incidence of fin-flicks (rapid flashing of dorsal, pelvic, or anal fin spines) was also noted, along with specific information on the identity of the fin-flicking fish and the subsequent behaviours of both individuals. This was recorded based on evidence from other families of fishes, that suggests that fin-flicks serve as a communicative signal^65^,^66^,^67^,^68^, and the potential importance of communication in reciprocal cooperation^36^,^37^. All occurrences of presumed flight behaviour (i.e. an individual rapidly abandoning its position followed by extensive swimming behaviour) were likewise recorded, specifying which individual initiated the behaviour, whether the fish’s visual fields were obstructed or not, and if the partner followed the flight. We observed 15 pairs each for three species (*S. corallinus, S. doliatus, S. vulpinus*), and 14 pairs for *S. puellus*, resulting in a grand total of 59 pairs, all of which were filmed for a period of 12 minutes. As only data points were included in which both pair members were in the video frame, and not visibly disturbed by the observer, the average number of non-independent data points (5-second intercepts) extracted per pair was 83.8 (±5.1 SE) for *S. corallinus*, 71.7 (±8.1 SE) for *S. doliatus*, 53.4 (±5.9 SE) for *S. puellus*, and 71.0 (±7.2 SE) for *S. vulpinus*.

To determine whether pair members sequentially alternated their roles between feeding bouts, we also counted the occurrence of alternating (A to B, B to A) and repeated (A to A, B to B) foraging bouts, with the latter also including instances where both pair members were feeding simultaneously (AB to A, AB to B, A to AB, B to AB, AB to AB). One foraging bout was defined as active foraging behaviour by either or both of the pair members (A, B, or AB) at a given 5-second intercept until it was interrupted by a different behaviour (or a change of the identity of the forager) at the next 5-second intercept. The next feeding bout commenced at the next 5-second intercept at which one of the individuals (or both) engaged in foraging behaviour. If the individual feeding during the previous bout was feeding again, repeated foraging behaviour was recorded. If the individual not engaged in foraging at the last 5-second intercept engaged in foraging, alternated foraging was recorded. Data were collected for all pairs in all species (n = 59).

In addition to the videos of pairing fish, 24 videos of solitary individuals in all four species (average of 6.0 ± 0.44 SE individuals per species; *S. corallinus: n* = 8; *S. doliatus: n* = 3; *S. puellus*: *n* = 7; *S. vulpinus: n* = 6; average observation period of 459.8 ± 41.6 SE seconds per individual) were collected and analysed to quantify the length of vigilance bouts, the number of bites per foray (foray defined as a continuous sequence of bites from the substratum), and the maximum substratum penetration depth during forays. Vigilance bouts refer to the duration (in s) over which a fish was observed in the assumed vigilance posture (a stationary ‘head-up’ position in the water column, exhibiting an angle >90°), without interruption by feeding or active swimming. The number of bites per foray was quantified as the number of consecutive bites taken by a fish without engaging in other behaviour such as vigilance (defined above). The penetration depth was estimated as the extent (in cm) to which a foraging fish penetrated into cracks in the substratum^61^. The same protocol was performed with 32 randomly selected videos of pairs (eight per species), where one haphazardly selected individual of the pair was selected for the duration of the video.

### Statistical procedures

We used linear and additive mixed effects models to separately analyse the angles of pair members during swimming and foraging (which, when combined, accounted for 92.8% of the behaviours displayed) for each species. We tested whether the angle of one individual in a pair (*θA*) was independent from predictor variables, including environmental factors (time of day, microhabitat complexity), or variables associated with the partner (the angle of the partner [*θB*], its height above the substratum, and the distance between pair members). For all analyses, pair ID was included as a random factor to account for non-independence of points taken from the same pair. For the data gathered during foraging activity, residual plots indicated non-linearity for *θA* as a dependent variable in all species. Thus, data were analysed using generalized additive mixed effects models (GAMMs) with a Gaussian error distribution and a cubic regression spline smoother, calculated by automatic cross-validation, for *θB* and *heightB* during foraging^69^. Due to temporal non-independence of behaviours (i.e. an individual might be more likely to assume an angle close to the angle from the previous data point), a temporal autocorrelation function was also added^70^. The analysis was repeated for *θB* as the dependent variable for all species. Variables included in the final model were selected using likelihood ratio tests, and model fits were assessed using residual plots. Angles during swimming were analysed in the same fashion but using generalized linear mixed effects models (GLMMs) with pair ID included as a random factor and incorporating a temporal autocorrelation coefficient. This was performed in order to demonstrate that the observed behavioural patterns during foraging are not simply a random behaviour, which is also present during other aspects of the fishes’ daily activity.

To test whether pair members alternated their roles more often than they performed the same role consecutively over the 12-minute observation period, occurrences of sequential changes in the identity of the foraging individual (either alternating or repeated foraging) were analysed. All transitions to or from bouts where both individuals were feeding simultaneously were assigned to be repeated in order to yield conservative estimates. The occurrence of alternating or repeated foraging bouts was analysed using four species-wise zero-inflated GLMMs with a negative binomial error distribution, using counts of alternated and repeated foraging events within pairs as dependent variables and pair ID as a random factor to account for the non-independence of data collected from the same pair. For all GLMMs, model fits were assessed using residual plots, all of which were satisfactory. For each pair, we also calculated the deviation from a balanced (0.5) proportion of 5-second intercept points at which individual A or B were foraging, and tested the overall distribution of feeding by pair members in each species against a balanced distribution using Pearson’s Chi-squared tests. The relative occurrence of fin-flicks during different behaviours (standardized as the number of fin-flicks per 5-second intercept during which a given behaviour was displayed) was analysed using a frequency test (Pearson’s Chi-squared test).

To examine potential behavioural differences between solitary and paired fish, solitary and paired individuals were compared for each species, separately, using GLMMs with the respective individual fitted as a random factor to account for non-independence of repeated measures for each fish. All data were modelled using a negative binomial error distribution as non-normality and overdispersion were detected during the modelling process. We tested the effects of the social status (solitary or paired) on the time spent in the vigilance posture (seconds), the number of consecutive bites per foray (bite counts), and the maximum extent to which individuals penetrated the substratum during foraging (cm). The value 1 was subtracted from the count dataset in order to prevent inaccurate estimates due to zero-truncation (i.e. as at least one bite was necessary to constitute a foray, there were no zeros in the count dataset, possibly resulting in inappropriate model estimates in a Poisson or negative binomial model^70^). For the time spent in vigilance posture, seconds were transformed to centiseconds to yield integer values. When the anterior structures of individuals were concealed due to penetration of the substratum, bites per foray were determined by the occurrence of caudal and pectoral fin-movements, which precede food acquisition in rabbitfishes^71^ (Supplementary Video S1). All analyses were performed using the software R and the packages *mgcv, nlme,* and *glmmADMB*.

## Results

**Figure 2:**
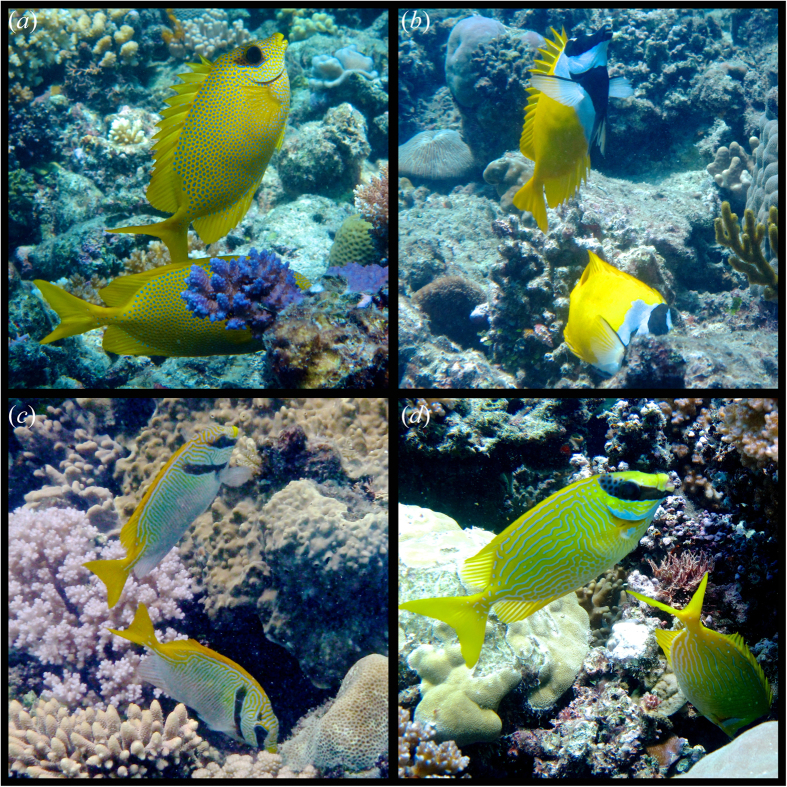
Foraging and vigilance postures in four species of pairing rabbitfishes. The foraging individual (in the head-down position) feeds in cracks and crevices in the substratum, while the vigilant individual is positioned in the water column with its head up. Note the obstructions to the visual field of the forager, suggesting high vulnerability to predation and the unobstructed field of perception of the vigilant fish. (**a**) *Siganus corallinus*, (**b**) *S. vulpinus*, (**c**) *S. doliatus*, (**d**) *S. puellus*. Photographs taken and owned by Jordan M. Casey, who gives permission for their publication under an Open Access license.

In all four rabbitfish species, when foraging, one pair member commonly assumed a stationary, upright position in the water column above the substratum (entailing an angle of 90° or larger), while the partner was feeding. The feeding individual often penetrated deep into cracks and crevices in the substratum with substantial obstructions to its visual field (Fig. 2, Supplementary Video S1). Possible flight responses (entailing rapid abandonment of vigilance position or foraging activity) were almost exclusively initiated by the individual positioned head-up in the water column (95.1% of instances), which always had an unobstructed field view of the surrounding environment. In contrast, at the onset of flights, the forager’s eyes were often not visible (15.7%), but it followed the fleeing individual in 94.1% of cases, suggesting that individuals in the water column were more vigilant than the forager and that information was rapidly communicated to the foraging fish. It is possible that some of the presumed flight responses were not due to the threat imposed by potential predators but rather in order to engage in territorial defence or simply to move on to another foraging location. However, the high density of predatory fish in the study area (and the presence of the observer as a potential threat) suggest that at least a proportion of the observed responses were associated with predator-oriented vigilance (Supplementary Video S2).

**Figure 3:**
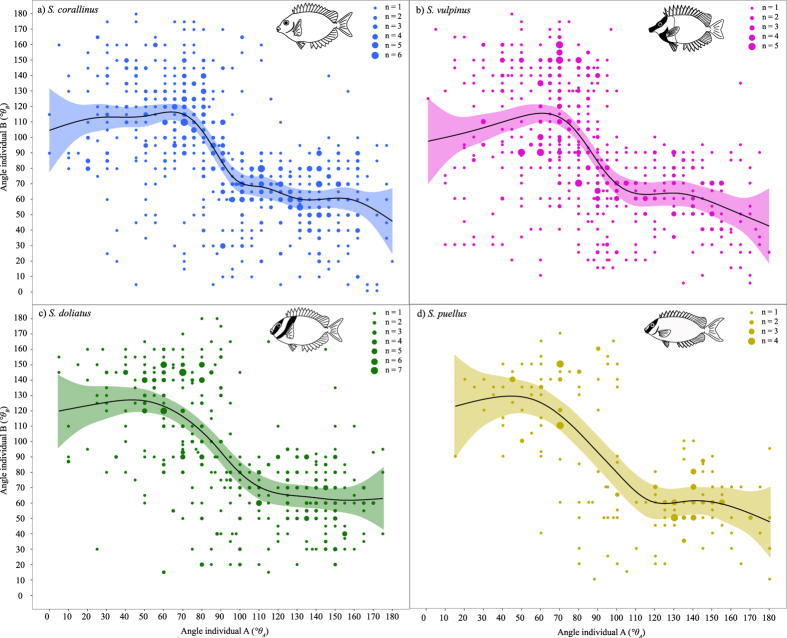
Graphical representation of coordination between foraging and vigilance in four species of rabbitfish pairs. Axes mark the angles of individuals in pairs (*θA, θB*), while each point represents the angle assumed at a given 5-second point intercept with the size varying according to the number of overlying points. The four predicted smoothed lines mark the predicted fits from generalized additive mixed effects models (GAMMs) and their upper and lower 95% confidence interval. In all species, data are predominantly spread between the upper left and lower right quartile of the plot. The smoothing function, fit by automatic cross-validation, suggests that individuals assume contrasting angles (<90° and >90°); however, the extent of the angle is negligible once a certain threshold is reached (~120° and 60°, respectively). The observed pattern was consistent among species. (**a**) *S. corallinus* (*n* = 15), (**b**) *S. vulpinus* (*n* = 15), (**c**) *S. doliatus* (*n* = 15), (**d**) *S. puellus* (*n* = 14). *n* = number of independent pairs represented in the plot. Fish illustrations are original drawings by Simon J. Brandl.

Pair members strictly coordinated their vigilance efforts, which is reflected by the angles assumed during foraging (Fig. 3). A low angle (=head down, foraging) in individual A (*θ*_*A*_) was complemented by a large angle (=head up, vigilant) in individual B (*θ*_*B*_) and *vice versa* in all four species. The GAMMs confirmed that pair members’ angles were non-independent and arranged in a contrasting manner (GAMM_AB_: *S. corallinus*: *edf* = 5.781; *F* = 11.23; *P* < 0.0001; *S. doliatus*: *edf* = 3.512; *F* = 11.94; *P* < 0.0001; *S. puellus*: *edf* = 3.802; *F* = 5.105; *P* = 0.0009; *S. vulpinus*: *edf* = 5.116; *F* = 4.438; *P* = 0.0005), suggesting that individuals take turns in being vigilant. The height of individual B likewise showed a significant inverse relationship with *θ*_*A*_ (GAMM_AB_: *S. corallinus*: *edf* = 3.865; *F* = 13.96; *P* < 0.0001; *S. doliatus*: *edf* = 3.716; *F* = 10.39; *P* < 0.0001; *S. puellus*: *edf* = 2.576; *F* = 2.576; *P* < 0.0001; *S. vulpinus*: *edf* = 3.668; *F* = 20.057; *P* < 0.0001), suggesting that an unobstructed field of perception (i.e. a large angle and a position high above the substratum) in one fish represents the best predictor for foraging (i.e. a low angle) in the partner (*S. corallinus*: *adj. R*^*2*^ = 0.428; *S. doliatus* = *adj. R*^*2*^ = 0.433; *S. puellus* = *adj. R*^*2*^ = 0.570; *S. vulpinus* = *adj. R*^*2*^ = 0.397). Except for *S. corallinus*, in which the time of day exhibited a small effect on the angle of individual A (*P* = 0.004; *adj. R*^*2*^ = 0.443), the angle and height of the partner were the only variables retained, as likelihood ratio tests indicated that the inclusion of other variables did not significantly improve the model fit. The analyses yielded similar results when repeated using *θ*_*A*_and height_A_ as predictors for *θ*_*B*_ (GAMM_BA_: *S. corallinus*: *adj. R*^*2*^ = 0.413; *S. doliatus* = *adj. R*^*2*^ = 0.462; *S. puellus* = *adj. R*^*2*^ = 0.529; *S. vulpinus* = *adj. R*^*2*^ = 0.327). In contrast to the angles during foraging, the angles of pair members while swimming showed a clear positive, linear relationship (GLMM_AB_: *S. corallinus*: *β* = 0.718; *t* = 22.56; *P* < 0.0001; *S. doliatus*: *β* = 0.761; *t* = 28.87; *P* < 0.0001; *S. puellus*: *β* = 0.644; *t* = 18.01; *P* < 0.0001; *S. vulpinus*: *β* = 0.673; *t* = 23.22; *P* < 0.0001), indicating that individual angles are non-independent and linearly synchronized during movement, with no other variables eliciting a significant effect in any of the four species (Supplementary Fig. S1). Thus, pairs of rabbitfishes travelled together synchronously, but performed contrasting, complementary roles during foraging, which were strongly coordinated with minimal overlap in vigilance behaviour.

**Figure 4:**
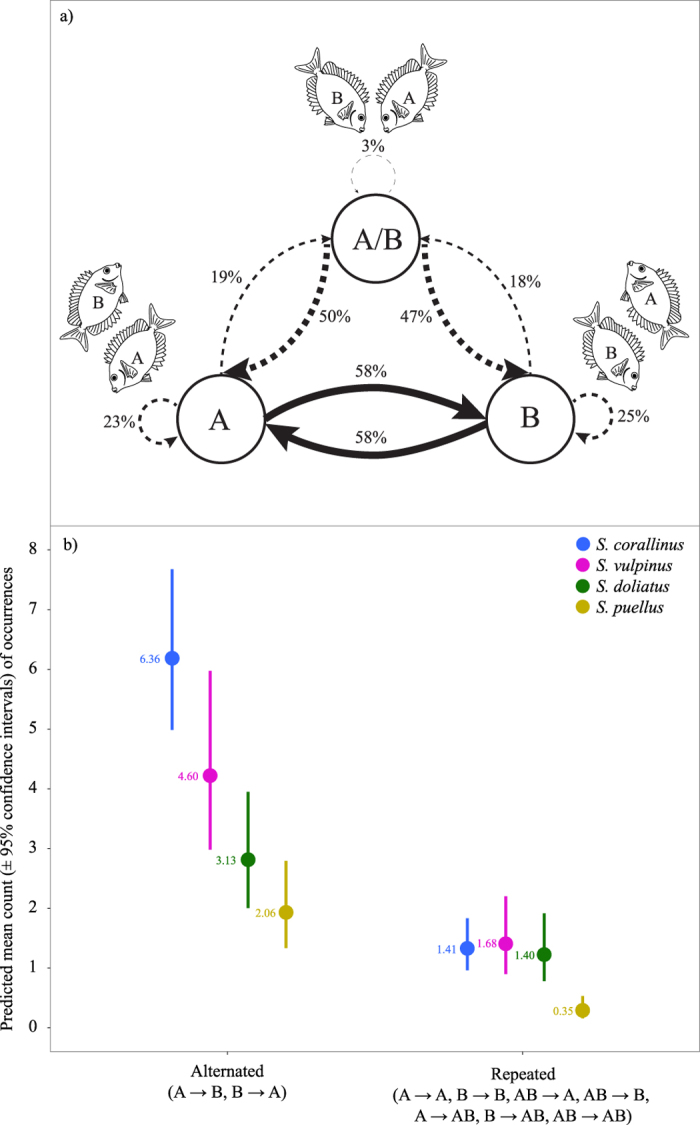
Patterns of alternated or repeated foraging bouts in pair members. (**a**) Schematic figure showing the percentage of different pathways pooled for all pairs in all species (*n* = 59). In cases where only one of the pair members is foraging (A or B), a subsequent foraging bout by the partner (B or A) is most common (58% and 58% of all sequential foraging bouts; solid arrows). In contrast, repeated foraging bouts were significantly less common (23% and 25%, respectively; dashed arrows) and so were changes to foraging bouts performed simultaneously by both individuals (19% and 18%, dashed lines). Foraging bouts performed by both pair members simultaneously were almost always succeeded by foraging bouts performed by a singular fish (50% A and 47% B). (**b**) The occurrence of alternated or repeated foraging bouts by pair members in all four species. Circles represent average values for a 12-minute period, predicted from zero-inflated generalized linear mixed effects models (GLMMs) with lines marking the upper and lower 95% confidence intervals. While the average number of both alternating and repeated foraging bouts varied among species as a function of the overall foraging activity, alternated foraging bouts (i.e. individuals taking turns) were significantly more common than repeated foraging bouts in all species (see text for statistical results). Observed values are provided to indicate model fits. Blue = *S. corallinus* (*n* = 15), magenta = *S. vulpinus* (*n* = 15), green = *S. doliatus* (*n* = 15), gold = *S. puellus* (*n* = 14). *n* = number of independent pairs represented in the plot. Fish illustrations are original drawings by Simon J. Brandl.

Across all pairing rabbitfish species examined, a vigilant individual (i.e., a stationary individual not engaged in foraging activity and exhibiting an angle >90°) was present during 82.6% of foraging activity (i.e. when at least one individual was feeding). When feeding, pair members alternated their roles significantly more often than they continued in the same role (Fig. 4a), with a higher proportion of alternated foraging bouts compared to repeated foraging bouts in all species (Fig. 4b; parameter estimates for repeated counts compared to alternated counts: *S. corallinus*: *β* = −1.538; *z* = −12.6; *P* < 0.0001; *S. doliatus*: *β* = −0.826; *z* = −5.61; *P* < 0.0001; *S. puellus*: *β* = −1.858; *z* = −7.99; *P* < 0.0001; *S. vulpinus*: *β* = −1.094; *z* = −7.70; *P* < 0.0001). The average deviation from a balanced proportion of feeding events per individual (0.5) was relatively small across pairs in all species (0.14), and within species, the distribution of feeding events was not statistically different from an expected balanced distribution (Pearson’s Chi-squared test; *S. corallinus*: χ^2^ = 2.419, *df* = 1, *P* = 0.120; *S. doliatus*: χ^2^ = 2.294, *df* = 1, *P* = 0.130; *S. puellus*: χ^2^ = 2.630, *df* = 1, *P* = 0.105; *S. vulpinus*: χ^2^ = 3.480, *df* = 1, *P* = 0.062), although these estimates have to be interpreted with care due to the relatively small sample size.

**Figure 5:**
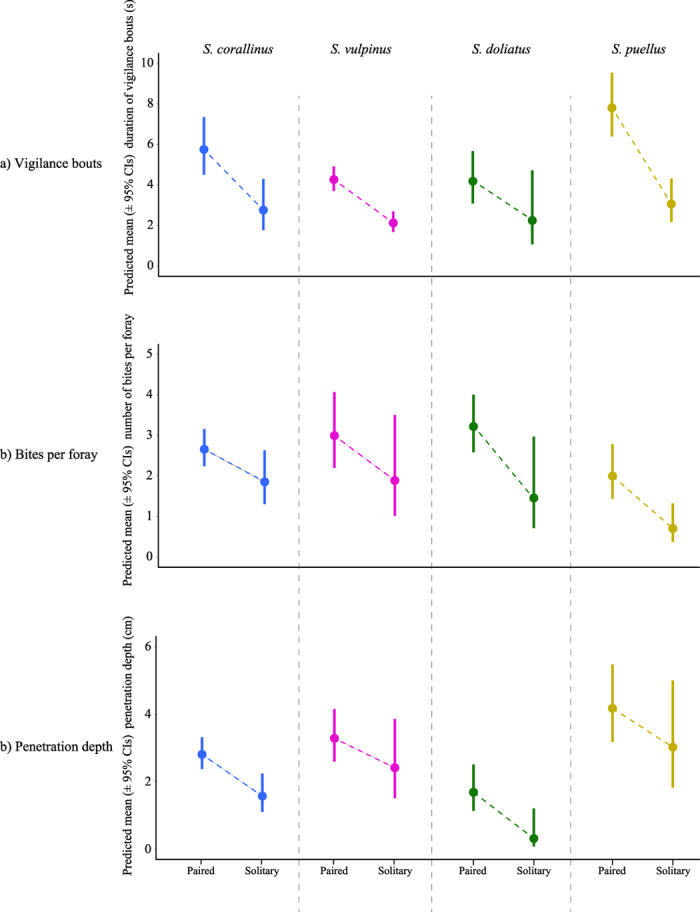
Behavioural differences between paired and solitary individuals in all four species. (**a**) Average predicted duration of vigilance bouts (±95% confidence intervals) in paired and solitary individuals. (**b**) Average predicted number of bites per foray (±95% confidence intervals). (**c**) Average predicted maximum penetration into cracks and crevices in the reef substratum during forays (±95% confidence intervals). All values were obtained from GLMMs performed separately on all four species. Blue = *S. corallinus* (pair *n* = 8; solitary *n* = 8), magenta = *S. vulpinus* (pair *n* = 8; solitary *n* = 6), green = *S. doliatus* (pair *n* = 8; solitary *n* = 3), gold = *S. puellus* (pair *n* = 8; solitary *n* = 7). *n* = number of independent individuals on which observations were performed.

The behaviour of solitary and paired rabbitfish individuals differed in all species (Fig. 5). Compared to solitary rabbitfishes, paired rabbitfishes exhibited significantly longer vigilance bouts except in *S. doliatus*, in which estimates followed the same trend but fell outside the α-level of 0.05 (parameter estimates for solitary individuals compared to paired individuals: *S. corallinus*: *β* = −0.733; *z* = −4.12; *P* < 0.0001; *S. doliatus*: *β* = −0.621; *z* = −1.64; *P* = 0.09; *S. puellus*: *β* = −0.934; *z* = −6.37; *P* < 0.0001; *S. vulpinus*: *β* = −0.695; *z* = −7.34; *P* < 0.0001). In all species, paired individuals took significantly more bites per foray (parameter estimates for solitary individuals compared to paired individuals: *S. corallinus*: *β* = −0.360; *z* = −2.48; *P* = 0.013; *S. doliatus*: *β* = −0.792; *z* = −2.29; *P* = 0.022; *S. puellus*: *β* = −0.935; *z* = −7.14; *P* < 0.0001; *S. vulpinus*: *β* = −0.461; *z* = −1.96; *P* = 0.05). In *S. corallinus* and *S. doliatus*, paired individuals penetrated deeper into the substratum than solitary individuals and while the same trend was visible in *S. puellus* and *S. vulpinus*, estimates in the latter two species were not statistically significant (parameter estimates for solitary individuals compared to paired individuals: *S. corallinus*: *β* = −0.578; *z* = −3.99; *P* < 0.0001; *S. doliatus*: *β* = −1.665; *z* = −2.58; *P* = 0.0099; *S. puellus*: *β* = −0.323; *z* = −1.75; *P* = 0.08; *S. vulpinus*: *β* = −0.308; *z* = −1.73; *P* = 0.084). Overall, differences between solitary and paired individuals were highly consistent among species. All species showed the same trends, varying only slightly in extent. The lack of significance in some variables may have arisen from small sample sizes in solitary individuals, which were both rare and exceptionally easily disturbed.

The relative frequency of fin-flicking differed significantly among behaviours (Pearson’s Chi-squared test: χ^2^ = 231.250, *df* = 3, *P* < 0.001), occurring significantly more often during foraging activity (i.e. when at least one of the fishes was engaged in foraging) and while displaying to other pairs (aggressive interaction) and hovering, while significantly less fin-flicks were performed during swimming. When one fish was in the vigilance position, while its partner was foraging, almost all observed fin-flicks were produced by the vigilant fish (92.4% of recorded fin-flicks during foraging activity) and subsequent actions (abandonment of current positions, switching positions, chasing) were taken by individuals on average 2.3 ± 0.2 seconds after the fin-flicking, suggesting that feeding individuals may respond to fin-flicks by the vigilant fish. This is further supported by the observation that 61.8% of all observed flight responses were preceded by fin-flicks.

## Discussion

In this study, we provide field-based observational evidence for a coordinated, cooperative vigilance system in four species of pairing rabbitfishes. Specifically, we demonstrate that during foraging, pair members strongly coordinate their positions: while one individual forages with its head down, its partner assumes an elevated, upright position in the water column, allowing for an unobstructed visual field to scan the surrounding environment. Paired fishes alternate frequently between foraging and the vigilance position. Compared to solitary individuals, individuals in pairs exhibit longer vigilance bouts than their solitary counterparts, but appear to benefit from the presence of the partner by exhibiting more consecutive bites per foray and deeper penetration into crevices in the substratum.

The posture assumed by one of the rabbitfishes while its partner is foraging closely matches reports in birds and mammals, where vigilant individuals are commonly identified by raised heads and/or elevated positions^72^,^73^,^74^,^75^, a behaviour that has only recently been reported for teleost fishes^62^. For rabbitfishes, the upright position is likely to favour vigilance as it potentially enables a greater ability to detect predators (and possibly also competitors) compared to foraging fishes, probably due to a less obstructed visual field^76^,^77^. The assumed angle may allow an unobstructed field of view while remaining close to the reef and the partner. While we are unable to demonstrate that the upright position serves primarily for the detection of predators, our observations and previous evidence suggest that scanning the surroundings for competitors, potential new partners, or food are of limited importance when compared to predator detection. While rabbitfishes do occasionally engage in aggressive behaviour with other pairs, these interactions are infrequent (<1% of the total behaviours observed in this study) and home-ranges of pairs are non-exclusive^58^, suggesting that scanning the surroundings for competitors may only play a minor role. In addition, pair bonds between rabbitfishes are relatively stable^57^,^58^, questioning the need to continuously look out for a new partner. Finally, the foraging strategy of rabbitfishes along with their dietary preferences for small and cryptic algae, sponges and cyanobacteria^61^,^63^, which require careful and close examination of concealed micro-habitats, make it unlikely that an elevated position will be beneficial for the detection of food. However, as we are unable to quantify the relative contributions of these various roles, we use the term ‘vigilance’ in its widest sense as being aware of the surrounding environment.

Whenever the pair was observed to rapidly abandon its positions (i.e. engaged in rapid directional swimming behaviour), the vigilant individual had an unobstructed view of the surrounding environment, while the visual field of the forager was often blocked by the reef substratum. Upon the vigilant individual abandoning its position (which was far more frequent than the forager abandoning its position first), the forager consistently trailed the vigilant individual, suggesting that foraging individuals reliably (94.1% of all cases) respond to actions or potential warning cues generated by the vigilant individual. While peripheral vision and social monitoring by the forager may facilitate the reaction to the vigilant partner’s behaviour^78^, the frequent occurrence of fin-flicks prior to abandoning the vigilance position may indicate intentional communication^79^,^80^,^81^. Although the significance of fin-flicks has not yet been investigated in rabbitfishes, fin-flicks are known to generate an acoustic signal in another family of reef fishes in which pairing is prevalent (f. Chaetodontidae^66^,^67^) and fin-flicks have been described as an important warning signal in other fish species^65^,^68^. Thus, although the role of fin-flicking remains to be determined in rabbitfishes, it seems likely that fin-flicks serve as a communication signal and that the forager is able to perceive these signals despite visual restrictions. Our findings that the vast majority of fin-flicks in rabbitfishes occurred in situations in which communication is beneficial (i.e. when one fish was foraging while the partner was vigilant or while displaying to other pairs) support a role of fin-flicks in in the maintenance of coordination between the forager and the vigilant fish.

Interestingly, the described scenario, in which foraging severely compromises vigilance while information is readily available from a vigilant conspecific, precisely matches the conditions under which coordinated vigilance should be favoured^37^,^76^. This is strongly supported by the angles rabbitfishes assume during foraging episodes where one fish’s angle and height above the substratum are the best predictors for complementary behaviour in the partner (Fig. 3). Clearly, pairs of rabbitfishes coordinate their positions during foraging and possibly do so through communication via fin-flicks.

Given this, the question then arises whether the coordinated behaviour in rabbitfish pairs represents a cooperative system based on by-product mutualism/pseudo-reciprocity^29^,^34^, or if rabbitfishes may satisfy the requirements of direct reciprocity. Several recent papers have emphasized the lack of evidence supporting the fundamental characteristics of direct reciprocity in animals^23^,^29^,^30^. These include: i) continuous cooperation between the same individuals, ii) behavioural adaptations to assist the partner, iii) adjustment of assistance provided according to received assistance, iv) cooperation not restricted to kin or potential mates, v) assistance entailing momentary net fitness costs to the assisting individual, and vi) cooperative behaviour being found in wild populations (after^23^).

For rabbitfishes, there is evidence supporting all of these requirements. (i) As rabbitfish pairs are stable and remain together for extended periods of time^57^,^58^, cooperation is likely to occur continuously between the same individuals. (ii) Our results show that individuals prolong the length of vigilance bouts in the presence of a partner. This may represent a behavioural adaptation to assist the partner, as paired fishes exhibited an increased number of bites per foray (*S. doliatus, S. puellus, S. corallinus*) or deeper substratum penetration (*S. corallinus, S. doliatus*), therefore increasing the likelihood of a higher overall food intake (as reported for pied babblers *Turdoides bicolor*^79^). (iii) Pair members frequently alternate between foraging and vigilance and the ratio between assistance provided and assistance received appears to be well balanced. While feeding was unevenly distributed in a few of the observed pairs, this may be a consequence of the length of observations, and a more balanced distributions may be observed if fish were monitored over an entire day. (iv) The observation that feeding within pairs is not generally skewed toward one individual suggests that cooperation is not solely based on male mate-guarding (as found in sleepy lizards, *Tiliqua rugosa*^82^), and the common occurrence of same-sex pairs in rabbitfishes^58^ suggests that cooperation is not limited to reproductive pairs^83^. Due to the reproductive strategies of reef fishes and their pelagic larval stage, cooperation limited to kin is also highly unlikely.

(v) Fitness costs may include predation risk and lost foraging opportunities during vigilance behaviour but such costs are inherently difficult to quantify^38^,^84^. As in other animals where mutual vigilance has been described as a potential cooperative system^72^,^79^,^85^,^86^, the vigilant individual in rabbitfish pairs is positioned above the underlying substratum with its head elevated, making vigilance beneficial for the overall awareness of the surroundings and therefore potentially self-serving rather than costly (provided predators selectively target foraging individuals). However, given the nutritive constraints of marine herbivory (or spongivory), which necessitate constant and intensive grazing, prolonged vigilance bouts are probably nutritionally costly rather than self-serving^87^. This is further supported by the low levels of simultaneous vigilance (both pair members hovering motionless above the substratum, 6.6% of behaviours across all pairs), which would indicate competition for vigilance in a scenario where predators preferentially target foragers^37^,^84^,^88^,^89^. Thus, while gaps in foraging activity associated with prolonged vigilance are likely to represent a significant cost, the deeper penetrations into crevices, as well as the higher number of bites per foray appear to be an intuitive reward for the partner. The lack of a clear difference between solitary and paired individuals in *S. puellus* and *S. vulpinus* in terms of penetration depth may point towards differences in the dietary preferences of these two species or their morphological adaptations. While *S. corallinus* and *S. doliatus* feed predominantly on filamentous and corticated red algae, food that is readily used by other herbivorous fish species^63^,^90^, only a few reef fishes feed on cyanobacteria, which are the main food source for *S. vulpinus*. Thus, the latter might be more readily available in more accessible microhabitats. In addition, *S. vulpinus* exhibits the morphological characteristics most suited for the exploitation of crevices among the four investigated species, suggesting that even solitary individuals might be able to penetrate into the substratum with no substantial obstructions to the visual field^59^. *S. puellus*, in turn, exhibits the most fusiform morphology among the examined species^59^, suggesting that quick escape from predators might play a bigger role in this species, possibly permitting solitary individuals to penetrate into the substratum despite the lack of a vigilant partner. However, given the relatively small sample size, the lack of significance in these comparisons should be interpreted with caution. These minor differences notwithstanding, there appear to be clear costs (vigilance bouts) and benefits (foraging efficiency) associated with cooperative vigilance in all four rabbitfish species examined. Finally, vi) all observations were conducted on the reef, indicating that the described vigilance system occurs in wild populations. Thus, the coordination of foraging and vigilance in rabbitfish pairs appears to satisfy all the basic requirements for reciprocal cooperation.

While we cannot hope to fully resolve the question of reciprocity with observational evidence alone, our findings are consistent with direct reciprocity. Thus, our study corroborates the tenor of several recent studies, which posit that reciprocity may be a lot more common under natural settings than when forced into the stringent rules of game-theoretical models^31^,^32^,^33^,^36^,^38^. Indeed, our results help us to understand why we may find reciprocity in animals, which lack the presumed cognitive requirements for reciprocity^23^. First, cooperative interactions among rabbitfishes are restricted to just one partner at a time. This alleviates frequently-cited cognitive issues arising from recognizing a range of individuals and remembering their previous actions in a large group of animals^13^,^24^,^27^ in order to repay for the behaviour of a previous partner, as has been posited for predator inspection and egg-trading in fishes^23^,^46^,^47^. Second, continuous foraging activity, immediate alternation, and the similar and simultaneous needs (food and safety) for both pair members in rabbitfishes prevent long time-lags between rounds in an IPD, as often found in, for instance, primates^33^,^91^. This again relaxes the need for complex cognitive abilities, such as memory, to underpin reciprocal cooperation^20^. Third, rabbitfishes cooperate continuously with the same partner over an extended period of time. There is now considerable evidence that such interdependence between social partners can foster high levels of cooperation in an IPD, as individuals do not systematically surrender to the short-term temptation of cheating on the partner^92^,^93^, therefore making tit-for-tat (or, more specifically ‘generous tit-for-tat’) a strategy with high levels of cooperation. Finally, our study provides preliminary evidence for the continuous exchange of social information between partners by means of fin-flicks and suggests that individuals quickly react to the behaviour displayed by the partner, as indicated by high levels of coordination^37^. Such elimination of discrete rounds in the IPD and the continuous exchange of information have been proposed as a major factor in favouring cooperation (either by coaction or reciprocity), as it lowers the cost for cooperating individuals^36^. Given the low levels of simultaneous vigilance observed in the present study, reciprocity appears more likely to operate in rabbitfishes than coaction, which would incline individuals to simply copy the partner’s behaviour^36^.

In summary, our study identifies pairing rabbitfishes as an intriguing group of animals in the context of reciprocity, cooperation, and cognition. Although limited to observational data, we provide strong evidence for a clear coordination of foraging and vigilance behaviour in pairs and demonstrate that pair members frequently alternate their foraging. We further show that rabbitfish pairs have the potential to satisfy all the basic requirements of reciprocity and discuss a range of conditions, which may favour reciprocal exchange in animals. Our evidence suggests that the complex cognitive and social skills, frequently assumed to be necessary for the evolution of direct reciprocity in animals, may be advantageous but, as in fishes, may not be essential.

## Additional Information

**How to cite this article**: Brandl, S. J. and Bellwood, D. R. Coordinated vigilance provides evidence for direct reciprocity in coral reef fishes. *Sci. Rep.* **5**, 14556; doi: 10.1038/srep14556 (2015).

## Supplementary Material

Supplementary Video S1

Supplementary Video S2

Supplementary Information
