# Supplementary material for: Coordinated vigilance provides evidence for direct reciprocity in coral reef fishes
Source: Sci Rep. 2015 Sep 25;5:14556. doi: 10.1038/srep14556 (PMC4585916; doi:10.1038/srep14556)
Supplement: Supplementary Information [file srep14556-s3.pdf]

Supplementary Information

Coordinated vigilance provides evidence for direct reciprocity in coral reef fishes

Authors

Simon J. Brandl[1,2] *, David R. Bellwood[1,2]

Affiliations

[1]ARC Centre of Excellence for Coral Reef Studies, James Cook University, Townsville, Queensland 4811, Australia

[2]College of Marine and Environmental Sciences, James Cook University, Townsville, Queensland 4811, Australia

*Corresponding author

Simon J. Brandl, James Cook University, Townsville, Queensland 4811, Australia, +61 7 4781 6657, simon.brandl@my.jcu.edu.au

Running head

Coordinated vigilance in reef fishes

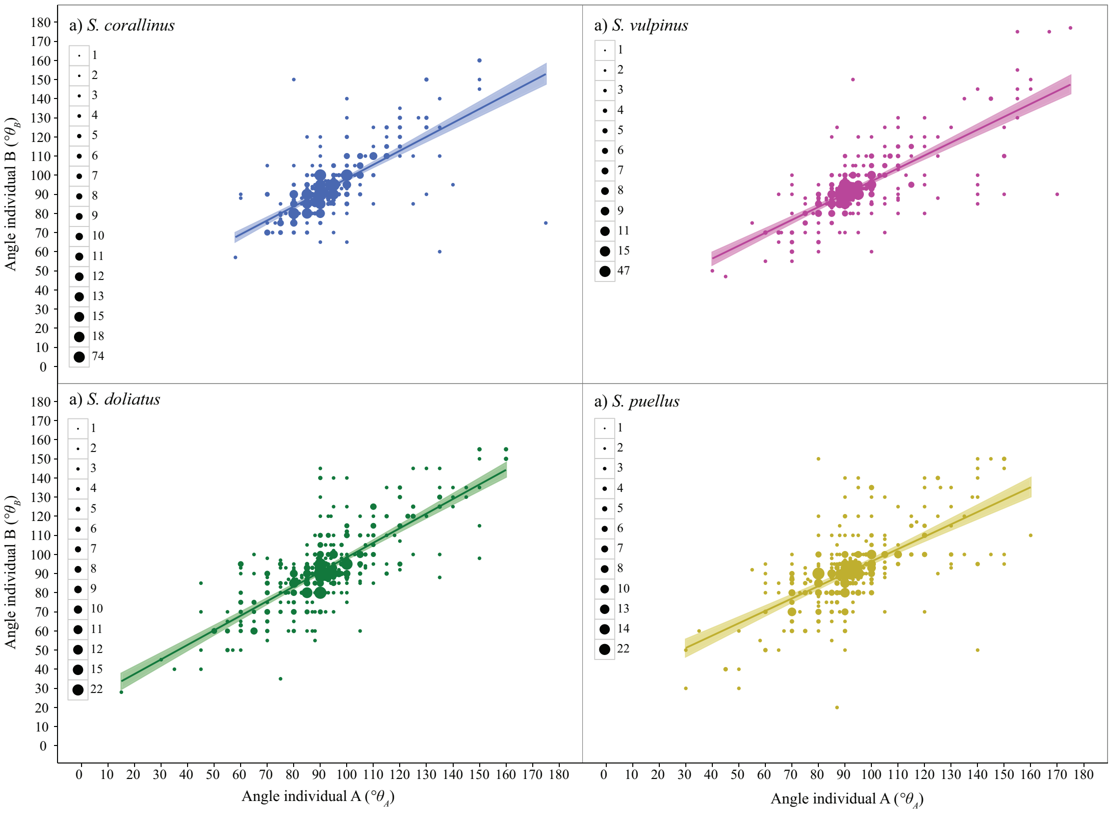

Figure S1: Angles exhibited by pair members during swimming behaviour. In all four species, angles are synchronized linearly, with the angle of individual B being the best linear predictor of the angle of individual A. The size of dots marks the number of overlying data points. Data represent repeated observations on 15 independent pairs in a) *S. corallinus*, b) *S. vulpinus*, c) *S. doliatus* and 14 independent pairs in d) *S. puellus*. Trendlines represent the predicted fit (± 95% confidence intervals) from GLMMs performed for each species separately.

Video S1: A pair of *S. doliatus* performing the described behaviour in which individuals alternate between foraging and a vigilance position, characterized by an upright position above the substratum.

Video S2: The reaction of a pair of *S. vulpinus* to the approach of a blacktip reef shark *Carcharhinus melanopterus*.
